# The Development of a Social Intelligence Test for Teenagers: A Pilot Study

**DOI:** 10.3390/children13040443

**Published:** 2026-03-25

**Authors:** Tracy Packiam Alloway, Daryn Argo, Sarah Gruskin, Baylee Comer

**Affiliations:** Department of Psychology, University of North Florida, Jacksonville, FL 32224, USA; darynargo@gmail.com (D.A.); n01461423@unf.edu (S.G.); n01423095@unf.edu (B.C.)

**Keywords:** social intelligence, emotional intelligence, social connectedness, friendship

## Abstract

**Highlights:**

**What are the main findings?**

• The Social Intelligence Test for Teenagers (SITT) has good internal reliability as measured by Cronbach’s alpha.
• The SITT has good convergent validity when compared to the Tromsø Social Intelligence Scale (TSIS).

**What are the implications of the main findings?**

• The SITT is a psychometrically sound tool to measure social intelligence in teenagers.
• It includes additional measures of social intelligence: social awareness and social perception.

**Abstract:**

Background/Objectives: Social intelligence refers to a person’s ability to understand and navigate social situations effectively. Teenagers who possess strong social intelligence are better equipped to build positive relationships, resolve conflicts, and make healthy decisions. Despite the importance of social intelligence, there are limited ways to measure this skill, especially during the teenage years. Thus, the aim of the present study was to develop and validate a scale that focuses on the following two aspects: social awareness and social perception. Methods: The reliability and validity of the Social Intelligence Test for Teenagers (SITT) was tested in teenagers aged between 14 and 18 years. Convergent validity was determined by the relationship between the SITT and the Tromsø Social Intelligence Scale (TSIS). Criterion validity was established by looking at the predictive relationship between the SITT and measures of social connectedness, empathy, and friendship. Results: The 19-item scale showed good internal reliability as measured by Cronbach’s alpha. The SITT mean score was significantly related to the TSIS Information Processing mean and the SITT Facial Expressions was significantly related to the TSIS Social Skills, even with age partialled out. Conclusions: The findings suggest that the SITT is a psychometrically sound tool to measure social intelligence in teenagers.

## 1. Introduction

Social intelligence refers to a person’s ability to understand and navigate social situations effectively [1]. It involves the capacity to perceive and interpret social cues, to communicate and empathize with others, and to regulate one’s own emotions and behavior in response to social interactions.

The teenage years are especially critical for the formation of social skills. During this developmental stage, teenagers are navigating complex social dynamics within their families, peer groups, and wider communities, as they begin to form their own identities and learn to interact with others in more mature ways.

### 1.1. Purpose of the Study

Given the relative scarcity of validated instruments for assessing social intelligence during the adolescent years, the primary objective of the present study was to design and evaluate a measurement scale tailored for this demographic. Social intelligence is conceptualized here as a multifaceted construct, to include communcation and conflict resolution skills. The theoretical background for the development of this scale draws on current research on how social intelligence is related to empathy, social connectedness, and friendship.

### 1.2. Social Intelligence and Empathy

The capacity for empathy is critical to social intelligence, as it mediates the relationship between emotional perception and the formation of high-quality social bonds. By validating their peers’ internal states, empathy promotes mutual trust, which contributes to relationship stability. In the present study, we included the Basic Empathy Scale (BES; [2]) to measure participants’ subjective sense of being understood within their social networks.

### 1.3. Social Intelligence and Social Connectedness

Social connectedness is the subjective sense of belonging and engagement within a community. Social intelligence and social connectedness form a reciprocal relationship, where social intelligence provides awareness into social dynamics and contextual nuances that can support social connectedness. Consequently, the present study includes the Social Connectedness Scale (SCS; [3]) to quantify the degree of participants’ perceived integration within their respective social networks.

### 1.4. Social Intelligence and Friendship

Social intelligence predicts the types of friendships formed during the teenage years. During the critical developmental window of adolescence, peer relationships emerge as a primary avenue for the practicing social skills. Adolescents who demonstrate high levels of self-disclosure and behavioral consideration are significantly more likely to cultivate resilient, supportive dyads that enhance overall psychological well-being. As such, the present study includes the Friendship Quality Scale (FQS; [4]) to assess the quality of these social bonds and the participants’ ability to adapt effectively within varied social settings.

## 2. Methods

### 2.1. Participants

Data were collected from 83 teenagers aged between 14 and 18 years (M = 15.67; SD = 1.42), from a private school in Northeast Florida (42.2% male; 42.2% female; 15.7% preferred not to respond). Of the respondents, 48.2% were Caucasian, 22.9% were African American, 3.6% were American Indian or Alaska Native, 3.6% were Asian, 1% were Native Hawaiian or Pacific Islander, and 20.5% self-identified as ‘Other’. Parental consent was obtained in line with ethical guidelines.

### 2.2. Measures

Social Intelligence Test: Items in the Social Intelligence Test for Teenagers included social situations involving friendship, bullying, and conflict resolution (*n* = 19; see Table 1 for a list of items). Items measuring an individual’s attention to the needs of peers during interpersonal exchanges were also included. This was measured using six facial expressions and six images of eyes. Faces were selected from an online database based on Paul Ekman’s research on the six universal emotions: fear, sadness, anger, joy, surprise, and disgust. The facial expressions were portrayed by the same female actor and were presented in black and white images. The six eye expressions were portrayed by the same female actor, in full color. Participants were only shown facial features between the nose and the forehead. All images were taken from the facial action coding system (FACS) website. For both the facial and eye expressions, participants were asked to identify the correct emotion that was portrayed from four options (generated from the universal emotions of: fear, sadness, anger, joy, surprise, and disgust). Emotions that were visually similar to the portrayed expression were included as distractors, for example, surprise and fear.

*Social intelligence*: Convergent validity was measured using the Tromsø Social Intelligence Scale (TSIS; [5]), which consists of 21 items capturing three dimensions; social skills, which measures communication skills such as active listening; social awareness, which measures the ability to engage in active behavior as appropriate to the situation; and social information processing, which measures the ability to understand verbal and nonverbal messages about relationships. It includes questions such as: “I know how my actions will make other people feel” or “I am good at getting on good terms with new people”. These items were assessed on a 7-point scale, ranging from (1) “*describes me extremely poorly*” to (7) “*describes me extremely well*”. ow scores indicate low social intelligence, while high scores indicate high social intelligence. This test has good internal reliability for the three dimensions, with Cronbach’s alpha coefficients as follows: social skills = 0.85; social awareness = 0.72; and social information processing = 0.79.

*Social connectedness*: This test is suitable for youths aged between 14 and 18 years of age and assesses the extent to which the individual feels connected to their social environment [3]. Eight negatively worded items from the Social Connectedness Scale–Revised were selected. Sample items include “I feel disconnected from the world around me”. Responses are rated on a six-point scale, ranging from (1) strongly agree to (6) strongly disagree. Scores are summed up and higher scores reflect more connectedness with others. Internal consistency with Cronbach’s alpha coefficient is 0.92.

*Empathy*: The Basic Empathy Scale is a 20-item scale developed by Jolliffe and Farrington [6], using teenagers as their sample. The scale includes items of cognitive empathy (*I can understand my friend’s happiness when she/he does well at something*) and affective empathy (*After being with a friend who is sad about something, I usually feel sad*). Participants respond on a 5-point scale, from 1 = Strongly Disagree to 5 = Strongly Agree. A meta review of 74 research articles identified Cronbach’s *α* for cognitive empathy as 0.81 and affective empathy as 0.81 [7].

*Friendships*: The Friendship Quality Scale developed by Bukowski, Hoza, and Boiving [4] was used to measure the quality of friendships. The scale comprises the following dimensions: companionship, conflict, closeness, help, and security. The 23 items are measured on a 5-point scale, with a neutral option, where (1) = *Not true at all* and (5) = *Really true*. Scores can range from 23 to 115, where lower scores indicate poor friendship quality and higher scores indicate high friendship quality. Cronbach’s alphas for the subscales are as follows: companionship = 0.72, conflict = 0.68, closeness = 0.76, help = 0.81, and security = 0.58.

### 2.3. Procedure

All participants completed the surveys online via an anonymous link generated by Qualtrics. They completed the surveys as a group, using school computers. The surveys were completed in a single sitting, lasting between 10 and 15 min. This was supervised by a classroom teacher.

## 3. Results

### 3.1. Data Analysis

The data were screened for univariate outliers. No individual’s score deviated greater or less than three standard deviations from the sample mean.

Data analysis was performed as follows. First, an exploratory factor analysis (EFA) was conducted to determine the factor structure of the Social Intelligence Test for the whole sample. Next, internal consistency reliability coefficients (Cronbach’s alpha) for each factor were estimated. Finally, correlational and regression analyses were conducted to explore convergent and criterion validity.

### 3.2. Reliability

Exploratory factor analysis:

The factor structure of all 18 items for the Social Scenarios dimension was tested using a Varimax rotation (eigenvalues > 1). Based on the correspondence of loadings in the pattern matrix across the sample, two items were eliminated. These items (*If your friends are making plans to hang out over the weekend and they UNINTENTIONALLY leave out your friend who is absent, what would you do?* and *If your friend invites you to try food from their culture (that is not your own), how comfortable would you feel going?*) were also excluded based on incongruences with the conceptual definitions of the relevant factors. The data yielded a four-factor solution, which explained 63.27% of the variance. Two items double-loaded on multiple factors and following common practice, the decision was made to retain each item in the factor on which it had the higher loading.

The first factor was labeled as expression and consisted of four items that captured how well the individual was able to express themselves in social situations. An example item is as follows: *How easy is it for you to express your thoughts and feelings to others?* Cronbach’s alpha for this factor was 0.66. The second factor captured three statements relating to bullying (*How likely are you to stand up to a bully?)*; Cronbach’s alpha for this factor was 0.70. The third factor included four statements relating to communication skills, such as: *When you interact with your friend, how well are you able to judge when to speak or keep quiet?*; Cronbach’s alpha for this factor was 0.67. The final factor related to five statements that addressed social conflict (*What would you do if your friends were laughing at someone else?*); Cronbach’s alpha for this factor was 0.58. Cronbach’s alpha for all remaining 16 items was 0.70. Cronbach’s alpha for the facial and eye expressions was 0.48. Due to Cronbach’s alpha being low (below the recommended cutoff of 0.70), these items are not included in the SITT mean score or in the subsequent analyses.

### 3.3. Validity

In order to establish convergent validity, a correlational analysis compared the subscales of the SITT, with the subscales from the Tromsø Social Intelligence Scale. The intercorrelations between the four SITT subscales were significant, suggesting good internal validity of the measures (*r*s ranged from 0.28 to 0.77), with the exception of the conflict subscale. As none of the zero-order correlations were higher than 0.80, multicollinearity was not a problem in this dataset [8].

However, these coefficients may have been inflated by the large age variation in this group. In order to adjust for this, a partial correlation analysis was calculated with age in years partialled out. These are shown in the upper triangle in Table 2. The intercorrelations between SITT subscales remained significant even after age was partialled out (*r*s ranging from 0.28 to 0.77). The within-construct coefficients were higher than the between-construct coefficients, suggesting good internal validity of the measures purportedly tapping the subscales of the SITT.

When comparing the SITT with the Tromsø Social Intelligence Scale (TSIS) for convergent validity, the SITT mean score was not significantly related to the TSIS mean or subscales. These patterns suggest that the SITT subscales capture a different aspect of social intelligence than the TSIS.

In order to investigate the criterion validity of the SITT in predicting social behaviors in the present sample, a series of hierarchical regression analyses was performed with sets of predictor variables entered separately for social connectedness, empathy, and friendship (see Table 3). It should be noted that this fixed-order hierarchical regression procedure is a highly conservative means of assessing unique relations when different variable sets are themselves highly correlated with one another, as in the present case. However, it does have the advantage of providing stringent tests of specificity of relations that are valuable for interpretating the data; any residual associations that do meet the criterion for statistical significance are therefore of particular note.

For social connectedness, communication skills (12%) was a significant predictor. For empathy, expression (5%) was a significant predictor. For friendship, communication kills (10%) was a significant predictor.

## 4. Discussion

This pilot study evaluated the psychometric properties of the Social Intelligence Test for Teens (SITT) to better understand how adolescents navigate their social worlds. Our findings provide evidence for the multidimensional nature of social intelligence and demonstrate that the SITT is a reliable and valid instrument for predicting key social outcomes in this demographic.

The exploratory factor analysis revealed a robust four-factor structure—*social expression, bully, communication skills, and conflict*—which collectively accounted for 63.27% of the total variance. The final 16-item scale demonstrated acceptable internal reliability (Cronbach’s alpha = 0.70). While the subscale reliability varied, notably with the *conflict* subscale (Cronbach’s alpha = 0.58), the correlations within the construct remained stable even when controlling for age. This stability indicates that the SITT captures core social competencies that are relatively consistent across the teenage years. Interestingly, the exclusion of facial and eye expression items due to low reliability (Cronbach’s alpha = 0.48) suggests that for modern teens, social intelligence may be more heavily rooted in the cognitive processing of social scenarios rather than the decoding of isolated non-verbal cues.

A key finding was the lack of a significant relationship between the SITT and the Tromsø Social Intelligence Scale (TSIS). This divergence suggests that the SITT captures a distinct aspect of social intelligence not covered by existing adult-centric measures, specifically tailored to the unique social pressures of adolescence, such as bullying and conflict management.

The predictive utility of the SITT was further confirmed through hierarchical regression analyses. Communication skills emerged as a critical predictor for social connectedness (12%) and friendship (10%). This underscores that knowing the “social timing” of interaction—such as when to speak or keep quiet—is a primary driver for building stable peer bonds. Social expression was a significant predictor for empathy (5%), which aligns with previous research [9,10] suggesting that the ability to articulate one’s internal state is closely linked to the capacity to resonate with the internal states of others.

### Limitations and Future Research

This research is the first to develop a psychometrically sound and theoretically driven scale to assess social intelligence in teenagers and how it predicts their friendships, empathy, and social connectedness. However, some limitations could be addressed. The participants in the present study were self-selecting, which may have affected the results. Future research could include clinical populations who are struggling to form meaningful social connections.

While this research is the first to develop a psychometrically sound, theoretically driven scale for adolescent social intelligence, several limitations warrant consideration. First, the current sample was self-selecting, which may introduce a volunteer bias that affects the generalizability of the results. Future research should aim to validate the SITT within clinical populations—specifically those struggling with neurodevelopmental or social-emotional challenges—to determine its diagnostic utility in identifying specific social deficits.

Additionally, the poor internal reliability of the facial and eye expression items and the moderate consistency of the social conflict subscale suggest areas for refinement. Adolescents in the digital age may process social information differently than previous generations; thus, future iterations of the SITT could explore more ecologically valid stimuli, such as video-based social scenarios or digital communication simulations, to better capture non-verbal and conflict-resolution competencies.

In summary, the development of the Social Intelligence Test for Teens (SITT) represents an important step in understanding youth social awareness and empathy. By identifying a robust four-factor structure—expression, bullying, communication skills, and conflict—this study demonstrates that social intelligence is a multidimensional construct with direct implications for a teen’s ability to form friendships and feel socially connected. Rather than viewing social intelligence as a static trait, this pilot study adopts the perspective that these competencies are plastic—capable of being refined through experiential learning and supportive social structures. By fostering such proficiencies, youths are better positioned to navigate interpersonal friction, make health-conducive decisions, and achieve superior outcomes in both academic performance and subjective life satisfaction.

## Figures and Tables

**Table 1:** Factor loadings > 0.40 from the exploratory factor analysis for the sample.

Items	Expression = 0.66	Bullying = 0.70	Communication = 0.67	Conflict = 0.58
How easy is it for you to express your thoughts and feelings to others?	0.74			
How confident are you that you can adapt in new social situations?	0.69			
How awkward do you feel around people who you are unfamiliar with?	0.66			
When working in a group, how easy is it for you to express your perspective?	0.52			
How likely are you to stand up to a bully who is harassing your friend?		0.77		
How likely are you to stand up to a bully who is harassing you?		0.76		
If you did stand up to a bully harassing your friend, how much anxiety would this give you?		0.59		
How often do you feel you are interrupting others during a conversation?			0.75	
When you interact with your FAMILY, how well are you able to judge when to speak or keep quiet?			0.74	
When you interact with your FRIEND, how well are you able to judge when to speak or keep quiet?			0.55	
When you interact with a group of people you are unfamiliar with, how well are you able to judge when to speak or keep quiet?			0.41	
If your group of friends were laughing at someone else, what would you do?				0.76
If you were to overhear your friend using an offensive slur, what would you do?				0.67
What do you feel is the best way to resolve conflict?				0.60
If you were to overhear a stranger using an offensive slur, what would you do?				0.46
If you made a perfect score on an exam, how would you act?				0.48

**Table 2:** Correlations between SITT and TSIS. Zero-order correlations in the lower triangle and correlations with age in years partialled out in the upper triangle.

	SITT Mean	Expression	Bully	Communication	Conflict	TSIS Mean	TSIS Social	TSIS Aware	TSIS IP
SITT mean	1	0.64 *	0.77 *	0.72 *	0.32 *	0.07	−0.09	−0.03	0.18
Expression	0.63 *	1	0.34 *	0.28 *	−0.04	0.17	0.06	0.11	0.16
Bully	0.77 *	0.34 *	1	0.41 *	0	−0.02	−0.12	−0.01	0.10
Communication	0.72 *	0.28 *	0.41 *	1	0.02	−0.04	−0.13	−0.18	0.09
Conflict	0.32 *	−0.04	0	0.02	1	0.10	0.01	0.05	0.13
TSIS Mean	0.07	0.17	−0.02	−0.04	0.10	1	0.76	0.63	0.72
TSIS Social	−0.09	0.06	−0.12	−0.13	0.01	0.76 *	1	0.51	0.29
TSIS Aware	−0.03	0.11	−0.01	−0.18	0.05	0.63 *	0.50 *	1	0.04
TSIS IP	0.18	0.16	0.09	0.09	0.13	0.73 *	0.30 *	0.04	1
	SITT mean	Adapt	Bully	Comm	Conflict	TSIS Mean	TSIS Social	TSIS Aware	TSIS IP

Note: * *p* < 0.01.

**Table 3:** Hierarchical regression analyses predicting social behaviors in the present sample.

	*R*^2^ Change	*F (df)*	*β*	*t*
Outcome: social connectedness				
SITT Communication	0.12	10.62 * (1, 80)	0.34	3.26 *
Outcome: empathy (BES)				
SITT Expression	0.05	4.16 * (1, 81)	0.22	2.04 *
Outcome: friendship (FQS)				
SITT Communication	0.10	9.45 * (1, 81)	0.32	3.07 *

Note: * *p* < 0.05.

## Data Availability

The original contributions presented in this study are included in the article material. Further inquiries can be directed to the corresponding author.
